# Quality of life assessment in amyloid transthyretin (ATTR) amyloidosis

**DOI:** 10.1111/eci.13598

**Published:** 2021-05-22

**Authors:** Alberto Aimo, Claudio Rapezzi, Federico Perfetto, Francesco Cappelli, Giovanni Palladini, Laura Obici, Giampaolo Merlini, Gianluca Di Bella, Matteo Serenelli, Mattia Zampieri, Paolo Milani, Roberto Licordari, Lucio Teresi, Nicolò Ribarich, Vincenzo Castiglione, Filippo Quattrone, Sabina De Rosis, Giuseppe Vergaro, Giorgia Panichella, Michele Emdin, Claudio Passino

**Affiliations:** ^1^ Institute of Life Sciences Scuola Superiore Sant’Anna Pisa Italy; ^2^ Fondazione Toscana Gabriele Monasterio Pisa Italy; ^3^ Cardiology Division University Hospital of Ferrara Ferrara Italy; ^4^ Maria Cecilia Hospital Cotignola Italy; ^5^ Regional Amyloid Centre Azienda Ospedaliero Universitaria Careggi Florence Italy; ^6^ Department of Internal and Experimental Medicine University of Florence Florence Italy; ^7^ Amyloidosis Research and Treatment Centre Fondazione IRCCS Policlinico San Matteo Pavia Italy; ^8^ Department of Molecular Medicine University of Pavia Pavia Italy; ^9^ Cardiology Division University of Messina Messina Italy; ^10^ Management and Healthcare Laboratory Institute of Management Scuola Superiore Sant’Anna Pisa Italy

**Keywords:** ATTR amyloidosis, PROMs, quality of life, scales, transthyretin

## Abstract

**Background:**

Amyloid transthyretin (ATTR) amyloidosis is caused by the systemic deposition of transthyretin molecules, either normal (wild‐type ATTR, ATTRwt) or mutated (variant ATTR, ATTRv). ATTR amyloidosis is a disease with a severe impact on patients’ quality of life (QoL). Nonetheless, limited attention has been paid to QoL so far, and no specific tools for QoL assessment in ATTR amyloidosis currently exist. QoL can be evaluated through patient‐reported outcome measures (PROMs), which are completed by patients, or through scales, which are compiled by clinicians. The scales investigate QoL either directly or indirectly, i.e., by assessing the degree of functional impairment and limitations imposed by the disease.

**Design:**

Search for the measures of QoL evaluated in phase 2 and phase 3 clinical trials on ATTR amyloidosis.

**Results:**

Clinical trials on ATTR amyloidosis have used measures of general health status, such as the Short Form 36 Health Survey (SF‐36), or tools developed in other disease settings such as the Kansas City Cardiomyopathy Questionnaire (KCCQ) or adaptations of other scales such as the modified Neuropathy Impairment Score +7 (mNIS+7).

**Conclusions:**

Scales or PROMs for ATTR amyloidosis would be useful to better characterize newly diagnosed patients and to assess disease progression and response to treatment. The ongoing ITALY (Impact of Transthyretin Amyloidosis on Life qualitY) study aims to develop and validate 2 PROMs encompassing the whole phenotypic spectrum of ATTRwt and ATTRv amyloidosis, that might be helpful for patient management and may serve as surrogate endpoints for clinical trials.

Amyloid transthyretin (ATTR) amyloidosis is caused by tissue deposition of full‐length and fragmented monomers of transthyretin (TTR), a tetrameric protein synthesized by the liver acting as a carrier for thyroxine (T4) and retinol‐binding protein.^1^ ATTR amyloidosis is a largely underdiagnosed disease, and it develops either as an age‐related phenomenon (wild‐type ATTR, ATTRwt) or as a result of *TTR* gene mutations (variant ATTR, ATTRv).^1^ Cardiac involvement is the main feature of ATTRwt amyloidosis, often associated with carpal tunnel syndrome and spinal stenosis. It is more common in men and has an estimated prevalence of 10% among individuals aged over 80 years.^2^ More than 130 potentially causative mutations have been described for ATTRv amyloidosis. Clinical presentation ranges from pure polyneuropathy (ATTR‐PN) to exclusive cardiomyopathy (ATTR‐CM), with a wide spectrum of mixed phenotypes. Among the most common mutations, V122I is related to a prevalent cardiac phenotype and is described in 3.4% of African Americans, while V30 M is characterized by a primarily neurologic phenotype and is endemic in several geographic areas such as Portugal and Japan.^1^

Patients with ATTR‐CM show a cardiac (pseudo) hypertrophy progressing towards heart failure (HF) with preserved ejection fraction (HFpEF).^3^, ^4^ Common signs and symptoms are shortness of breath, leg swelling, fatigue and eventually cachexia.^4^, ^5^, ^6^ Intracardiac conduction disorders and arrhythmias, particularly atrial fibrillation, are also common. In patients with ATTR‐PN, sensory, motor and autonomic fibres are involved. Typical manifestations include paresthaesia, hypoesthaesia, numbness and pain progressing from hands and feet to arms and legs. In more advanced stages, large‐fibre neuropathy can develop, eventually leading to wheelchair or bed confinement. Autonomic dysfunction can manifest at different disease stages causing arrhythmias and orthostatic hypotension, as well as gastrointestinal, genital and urinary disturbances. Typical symptoms are palpitations, fatigue, postural dizziness, blurred vision, syncope, slow digestion, post‐prandial nausea, vomit, dysuria, urinary retention, pollakiuria, stress incontinence and erectile dysfunction.^7^

Amyloid transthyretin amyloidosis has a severe impact on patients’ health and quality of life (QoL), as confirmed by many observational studies (Table 1).^8^, ^9^, ^10^, ^11^ This may be related to the long time usually required to establish the diagnosis, the slow disease progression, the high frequency of systemic involvement and age‐related comorbidities, together with the limited therapeutic options. Furthermore, patients with ATTRv amyloidosis often worry about disease transmission to their children and grandchildren. Despite its relevance, there are currently no specific tools for QoL assessment in ATTR amyloidosis, which nonetheless would be extremely useful to better characterize patients at baseline and to assess disease progression and the response to therapies.

**TABLE 1:** Assessment of quality of life (QoL) in phase 3 clinical trials

Drug	Author, year	Design (phase 3 trials)	Population	PROMs	Other tools for QoL assessment	Time of the assessment
Tafamidis	Maurer, 2018 (ATTR‐ACT)	Multicentre, double‐blind, placebo‐controlled trial (completed) 2:1:2 randomization to oral tafamidis 80 mg daily, tafamidis 20 mg daily or placebo for 30 mo	ATTR‐CM (wt and v) Pooled tafamidis n = 264 Placebo n = 177	KCCQ	6MWT	Baseline Month 30
Patisiran	Adams, 2018 (APOLLO)	Multicentre, double‐blind, placebo‐controlled trial (completed) 2:1 randomization to iv patisiran (0.3 mg/kg) or placebo every 3 wk for 18 mo	ATTRv‐PN n = 225 (126 with cardiac disease)	QoL‐DN COMPASS‐31	mNIS+7_Alnylam_ R‐ODS 10‐metre walking test PND	Baseline Month 30
Inotersen	Benson, 2018 (NEURO‐TTR)	Multicentre, double‐blind, placebo‐controlled trial (ongoing) 2:1 randomization to weekly sc inotersen (300 mg) or placebo for 64 wk	Stage 1‐stage 2 ATTRv‐PN n = 172 (108 with cardiac disease)	QoL‐DN	mNIS+7_Ionis_	Baseline Week 66
Revusiran	Judge, 2020 (ENDEAVOUR)	Multicentre, double‐blind, placebo‐controlled trial (completed) 2:1 randomization to sc revusiran (500 mg) or placebo for 18 mo	ATTRv‐related cardiomyopathy n = 206 patients	KCCQ	6MWT NYHA class	Baseline Month 18
Vutrisiran (ALN‐TTRSC02)	NCT03759379 (HELIOS‐A)	Multicentre, double‐blind, placebo‐controlled trial (ongoing) Vutrisiran (500 mg) or iv patisiran (0.3 mg/kg) for 18 mo	ATTR (v) n = 164	QoL‐DN	mNIS+7_Alnylam_ R‐ODS 10‐metre walking test	Baseline Month 9 Month 18
AKCEA‐TTR‐LRx (IONIS 682884)	NCT04136171 (CARDIO‐TTRansform)	Multicentre, double‐blind, placebo‐controlled trial (ongoing) Parallel randomization to sc AKCEA‐TTR‐LRx (45 mg) once every 4 wk or placebo for 120 wk	ATTR (wt and v) cardiomyopathy n = 750	KCCQ	6MWT	Baseline Week 61 Week 120
AG10	NCT03860935 (ATTRIBUTE‐CM)	Multicentre, quadruple‐blind, placebo‐controlled trial (ongoing) Parallel randomization to oral AG10 800 mg twice daily for 30 mo	ATTR‐related cardiomyopathy (wt and v) n = 510	KCCQ	6MWT	Baseline Month 12 Month 30

Abbreviations: 6MWT, 6‐min walking test; ATTR, amyloid transthyretin amyloidosis; ATTR‐CM, ATTR cardiomyopathy; ATTR‐PN, ATTR polyneuropathy; ATTRv, variant ATTR; ATTRwt, wild‐type ATTR; COMPASS‐31, COMPosite Autonomic Symptom Scale 31 questionnaire; KCCQ, Kansas City Cardiomyopathy Questionnaire; NIS, Neuropathy Impairment Score; NYHA, New York Heart Association; PND, PolyNeuropathy Disability score; PROMs, patient‐related outcome measures; QoL‐DN, Norfolk QoL‐Diabetic Neuropathy questionnaire; R‐ODS, Rasch‐built Overall Disability Scale; SF‐36, 36‐Item Short Form Survey.

For this review, clinical studies on ATTR amyloidosis were searched on the clinicaltrials.gov website using the following keywords: “amyloidosis”, “transthyretin amyloidosis” or “ATTR amyloidosis” (field: “condition or disease”; last update on 24 April 2021). We selected all phase 2 or phase 3 studies on ATTR amyloidosis, either completed or ongoing (n = 44). The sources were the Outcomes section of the clinicaltrials.gov website and, when available, the corresponding publications. The methods for assessing QoL were extracted independently by 2 authors (LT and NR), and controversies were solved by discussion with a third author (AA). These methods were classified into measures of general health status and disease‐specific measures of cardiac involvement or neuropathy. Reporting of the study conforms to broad EQUATOR guidelines.^12^

## 1 PROMS VS SCALES

Patients’ QoL can be evaluated through 2 different approaches: (a) patient‐reported outcome measures (PROMs), which are completed by patients; and (b) scales, which are compiled by clinicians and investigate QoL either directly or indirectly, that is by assessing the degree of functional impairment and limitations caused by the disease.^13^ PROMs collect subjective information, but they have been proved to be noninferior to clinical scales in evaluating QoL and predicting survival.^14^ While scales require office visits, PROMs can be administered in various forms including self‐administration, possibly using an online platform. This avoids the risk of biases related to the presence of a professional interviewing or observing the patient.^14^ While scales measure functional parameters over time, PROMs focus on patient perception of the disease rather than the disease itself. For all these reasons, PROMs are becoming the method of choice to assess patients’ QoL.

Scales and PROMs can either explore the general health status or be disease‐specific. General health status measures may allow comparisons between different disorders, although this comparison may be misleading since different diseases have a heterogeneous impact on the same life activities (eg in simple performances like getting dressed HF patients may be hindered by fatigue and dyspnoea, while patients with Parkinson's disease may be impeded by tremor).^15^ Disease‐specific QoL measures focus on the most important aspects of a certain illness; therefore, they are potentially responsive to treatment‐related changes and can show differences between alternative therapies.^15^ Clinical trials on ATTR amyloidosis have used either generic or specific measures originally created for other cardiac or neurologic disorders (Tables 1 and 2).

**TABLE 2:** Assessment of quality of life (QoL) in observational studies

Study	Author, year	Design	Population	QoL metric
NCT00628745 (THAOS)	Coehlo, 2013 Maurer 2016	Multicentre, prospective longitudinal observational study	ATTR (wt and v)	EQ5D‐5L + EQ‐VAS
			n = 952; n = 2530	QoL‐DN
NCT01604122	Stewart, 2018	Multicentre, prospective cross‐sectional study	ATTR (wt and v)	EQ5D‐3L
			n = 60	SF‐12
				WPAI‐SH
				HADS
				QoL‐DN
				KCCQ
				ZBI
	Lane, 2019	Monocentric, prospective longitudinal observational study	ATTR (wt and v)	KCCQ (time assessment: 12 and 36 mo)
			n = 1034	

Abbreviations: EQ5D‐5L, Euro QoL 5‐Dimensions 5‐Levels Questionnaire; EQ‐VAS, Euro QoL Visual Analogue Scale; HADS, Hospital Anxiety and Depression Scale; KCCQ, Kansas City Cardiomyopathy Questionnaire; PROMs, patient‐related outcome measures; QoL‐DN, Norfolk QoL‐Diabetic Neuropathy questionnaire; SF‐12, 12‐Item Short Form Survey; WPAI‐SH, Work Productivity and Activity Impairment Questionnaire: Specific Health Problem; ZBI, Zarit Burden Interview.

## 2 MEASURES USED TO ASSESS QOL IN PHASE 2 OR PHASE 3 CLINICAL TRIALS ON ATTR AMYLOIDOSIS

### 2.1 Measures of general health status

The *36‐Item Short Form Survey (SF‐36)* is the most used PROM to assess health‐related QoL. It consists of 36 questions covering 8 domains: 4 on physical health and 4 on mental health, with a focus on the last 4 weeks. A grade from 0 to 100 is assigned to each domain, with lower scores associated with a worse QoL. The average of these 8 domains is the total SF‐36 score.^16^ A shorter questionnaire with 12 items (SF‐12) is also available, with similar accuracy to the complete score.^17^

The *Euro* *QoL 5‐Dimensions 3‐Levels (EQ5D‐3L) questionnaire* is another PROM evaluating health‐related QoL. Patients are asked to rate 5 dimensions (mobility, self‐care, usual activities, pain or discomfort, and anxiety or depression) from 1 to 3, with 1 corresponding to ‘no problems’, 2 to ‘some problems’ and 3 to ‘severe problems’. This assessment is usually integrated by the *Euro* *QoL* *Visual Analogue* *Scale* *(EQ‐VAS)*, whereby the patient is asked to point his/her current health status on a 20‐cm vertical scale ranging from 0 (‘the worst health you can imagine’) to 100 (‘the best health you can imagine’).^18^

The *Work Productivity and Activity Impairment Questionnaire: Specific Health Problem (WPAI‐SH)* is a questionnaire including 6 items (current employment status, hours missed from work due to the disease, hours missed from work for other reasons, hours effectively worked, degree of health‐related impairment on daily activities) concerning the 7 days before the questionnaire administration, with different scales for each item and higher scores indicating a greater impairment.^19^ The *Hospital Anxiety and Depression* *Scale* *(HADS)* is a questionnaire with 7 items for anxiety and 7 for depression. Each item is given a score from 0 to 3, with higher scores for more severe symptoms. Each 7‐item subgroup score is summed up, resulting in 2 separate subscale scores from 0 to 21.^20^

The *Karnofsky* *Performance Status* *Scale* was proposed in 1947 for cancer patients and evaluates general well‐being and functional impairment in daily life, with an alphabetic and a numeric score system. Patients are classified into group A if they are ‘able to carry on normal activity and to work’, group B if they are ‘unable to work’ and group C if they are ‘unable to care for self’. A further evaluation is made with 11 categories and a score from 0 to 100, with 0 equals to death and 100 equals to ‘able to carry on normal activity and to work’.^21^

The *Patient Global Assessment (PtGA)* is a score proposed for evaluating pain in rheumatoid arthritis during follow‐up with a simple question. At the baseline, the question is ‘In general, how do you feel today?’ and the answer is rated from 1 (excellent) to 5 (poor). At follow‐up visits, the question is ‘How do you feel today as compared to when we talked with you at your last clinic visit for this study?’ and the answer is rated from 1 (markedly improved) to 7 (markedly worsened).^22^

The *10‐metre walking test* is a performance measure that can be used to assess walking ability and autonomy at different speed over a short distance, and has been evaluated as a secondary end point in APOLLO^23^ and HELIOS‐A trials (NCT03759379).

The *Zarit* *Burden Interview (ZBI)* is one of the most used questionnaires for caregivers, which has been employed also in the setting of ATTR amyloidosis.^10^ It investigates psychological suffering, financial difficulties, shame, guilt and difficulties in social and family relationships related to caregiving. The most common version has 22 items, each consisting of a 5‐point scale from 0 (never) to 4 (nearly always).

### 2.2 Disease‐specific measures: cardiac involvement

The *Kansas City Cardiomyopathy Questionnaire (KCCQ)* is a common tool for QoL assessment in trials on ATTR‐CM. The KCCQ is a 23‐item PROM evaluating the impact of HF.^24^ The KCCQ includes 6 domains referring to the 2 weeks before questionnaire administration: symptoms (frequency and severity of fatigue, shortness of breath or leg swelling), symptom stability, physical functioning, social limitation, QoL and self‐efficacy (perceived ability of preventing or managing HF decompensations). Each domain ranges from 0 to 100 with higher scores reflecting a better health status. Two summary scores can be calculated: a clinical summary score (evaluating symptoms and physical functioning) and an overall summary score.^24^

The *New York Heart Association (NYHA) classification* has been commonly used to categorize HF patients enrolled in phase 3 trials on ATTR‐CM, most notably the ATTR‐ACT.^25^ Attribution of the NYHA class is simple but relies on patient's reliability and physician's evaluation. Therefore, a change in NYHA class is rarely used as an end point in clinical trials.

The *6‐minute walking test* is a more objective metric of the cardiorespiratory fitness, and changes in walking distance may serve as an end point in clinical trials on ATTR amyloidosis.^23^, ^25^, ^26^ Nonetheless, the 6‐minute walking distance correlates with peripheral neuropathy rather than the severity of cardiac involvement in patients with ATTRv amyloidosis.^27^

We have not found any phase 2 or phase 3 studies on ATTR amyloidosis employing the Minnesota Living with Heart Failure Questionnaire, which is a commonly used PROM to assess QoL in patients with HF.^28^

### 2.3 Disease‐specific measures: neuropathy

Several measures have been proposed to estimate the severity of neurological impairment in patients with ATTR amyloidosis, which in turn is closely correlated with patient QoL.

The *polyneuropathy disability (PND) score* can be used to classify ATTR‐related neuropathy in the following stages: I, sensory disturbances with preserved walking capacity; II, impaired walking capability but no need for a stick or a crutch; IIIa, need for a stick or a crutch for walking; IIIb, 2 sticks or crutches required for walking; and IV, confinement to a wheelchair or bed. The PND score is a gross classification, but still useful as a first approach to assess neuropathy severity.^5^

The *Neuropathy Impairment Score (NIS)* was originally created for neurologic assessment in diabetes. The NIS is a composite score that quantifies muscle weakness, muscle stretch reflex and sensory loss, and it is named NIS‐LL (lower limbs) when focused on lower limbs functions. The NIS+7 adds nerve conduction measures in the tibial, peroneal or sural nerves, vibration detection threshold of the great toe and heart rate variability in response to 1‐minute deep breathing. Two modified NIS+7 scores have been developed for ATTR amyloidosis, namely the mNIS+7_Alnylam_ (range: 0‐304)^23^ and the mNIS+7_Ionis_ (range: 0‐346.3).^26^ The Summated 7 Score for large nerve fibre function and the Summated 3 Score for small nerve fibre function are NIS subscales composed only by clinical and instrumental neurological tests.

The *Kumamoto Neurologic* *Scale* is a 14‐item score exploring sensory impairment, autonomic disfunction, muscle weakness and visceral organ impairment. This tool ranges from 0 to 96, with higher scores denoting a worse limitation. It is not usually used alone, but in association with more detailed scores such as the NIS.^27^, ^29^

The *Rasch‐built Overall Disability* *Scale* *(R‐ODS)* is a 24‐item scale created to assess limitations in activities and social functioning in patients with Guillain–Barré syndrome or similar disorders. A new version with 34 items has been made available: the *Familial Amyloid Polyneuropathy specific* *Rasch‐built Overall Disability* *Scale* *(FAP‐RODS)*.^30^

The *Norfolk* *QoL‐Diabetic Neuropathy (QoL‐DN) Questionnaire* was originally designed for diabetic neuropathy^31^ and has been recently validated for V30 M ATTR‐PN.^32^ It has been used in many clinical trials as APOLLO,^23^ HELIOS‐A (NCT03759379) and NEURO‐TTR.^26^ It is a 35‐item questionnaire assessing physical functioning, daily life activities, symptoms, small‐fibre neuropathy and autonomic neuropathy. The answers are rated on a 5‐point scale, with higher scores indicating a worse status. The 5 domains can be considered alone or together as a sum evaluating the total quality of life.

The *COMPosite* *Autonomic Symptom* *Scale* *31 (COMPASS‐31) Questionnaire* was originally developed for patients with small‐fibre polyneuropathy, with 31 items organized in 6 domains investigating orthostatic intolerance, vasomotor, secretomotor, gastrointestinal, bladder and pupillomotor activities.^33^

## 3 LIMITATIONS OF CURRENT TOOLS FOR QOL ASSESSMENT IN ATTR AMYLOIDOSIS

Amyloid transthyretin amyloidosis affects several organs and systems, and symptoms vary among patients and at different times. Evaluating disease burden is challenging because there is no single measure or set of measures able to capture the full spectrum of symptoms. Nonetheless, filling many questionnaires would be burdensome for patients, and different measures are partially overlapping and redundant (Table 3).

**TABLE 3:** Domains explored and limitations of quality‐of‐life measures used in amyloid transthyretin amyloidosis (ATTR)

SCORE	KCCQ	PND	KUMAMOTO	R‐ODS	QOL‐DN	NIS	COMPASS‐31	SF‐36	EQ5D‐3L	HADS	WPAI‐SH	Karnofsky	PGA
Domains													
Cardiac	Yes		Yes										
Gastro‐intestinal			Yes		Yes		Yes						
Neuropathies		Yes	Yes	Yes	Yes	Yes							
Autonomic function			Yes		Yes	Yes	Yes						
Genera health status								Yes	Yes			Yes	Yes
Physical functioning	Yes	Yes		Yes	Yes			Yes					
Mental health								Yes		Yes			
Impact on work					Yes			Yes			Yes		
PROMs or scale?	PROMs	Scale	Scale	PROMs	PROMs	Scale	PROMs	PROMs	PROMs	PROMs	PROMs	Scale	PROMs
Original application	CHF patients	FAP	FAP Val30Met	Immune‐mediated peripheral neuropathies	Diabetic neuropathy	Diabetic neuropathy	Autonomic disorders	Generic patients	Generic patients	Generic outpatients	Workers	Cancer patients	Rheumatoid arthritis
Limitations													
Validated for ATTR	Yes	Yes	Yes	Yes	Yes	Yes							
Other limitations	Low reliability of self‐efficacy subscale	Broad categories (>5 y to change category	No psychometric analysis	Tested only in Portugal and in FAP Val30Met	Domains with overlapping scores	Ceiling effect	Exclusive assessment of dysautonomia	Sleep quality not assessed	Poor correlation with clinics in HFrEF	Only assesses anhedonia	Useless in unemployed	Difficulty in scoring aggregations, there are more possible classes than those provided.	No standardized phrasing
	QoL subscale redundancy			Emotional sphere and symptoms severity not assessed	No content validation	No dysautonomia subscale		Floor effect	Not studied in HFpEF		Tested only in white‐collar jobs		
						Complex score			Ceiling effect				

Abbreviations: CHF, chronic heart failure; COMPASS‐31, COMPosite Autonomic Symptom Scale 31 Questionnaire; EQ5D‐3L, Euro QoL 5‐Dimensions 3‐Levels Questionnaire; HADS, Hospital Anxiety and Depression Scale; Karnofsky, Karnofsky Performance Scale; KCCQ, Kansas City Cardiomyopathy Questionnaire; NIS, Neuropathy Impairment Score; PGA, Patient General Assessment; PND, PolyNeuropathy Disability score; PROMs, patient‐related outcome measures; QoL‐DN, Norfolk QoL‐Diabetic Neuropathy Questionnaire; R‐ODS, Rasch‐built Overall Disability Scale; SF‐36, 36‐Item Short Form Survey; WPAI‐SH, Work Productivity and Activity Impairment Questionnaire: Specific Health Problem.

### 3.1 Measures of general health status

The *SF‐36* does not measure sleep quality, which is an important indicator of health in amyloidosis, as many patients experience sleep‐disordered breathing.^34^, ^35^ Furthermore, a ‘floor effect’, that is the inability to stratify patients with low QoL, has been demonstrated for the SF‐6D, a questionnaire developed from the SF‐36.^36^

The *EQ5D‐3L* has been studied in patients with HF with reduced EF (HFrEF), in whom it showed a poor correlation with clinical improvements,^37^, ^38^ while its efficacy in HFpEF has been less explored. Furthermore, a ceiling effect can be expected, as each question has only 3 possible answers. This problem has been partially overcome with the introduction of a 5‐level version (*EQ5D‐5L)*, which should be preferred in future trials.^38^, ^39^

The *WPAI‐SH* cannot be used to assess unemployed patients, and should not be used to evaluate patients doing jobs that entail moderate to intense physical activity.^20^

The *HADS* has been criticized because it assesses only the anhedonic domain of depression, hence may fail to capture other manifestations of depression.

The Karnofsky Performance Status Scale lacks strict criteria for patients’ stratification, resulting in an important variability when different physicians evaluate the same patient.^21^ For example, it is not clear what should be considered ‘a normal activity’ or an ‘active work’. The PtGA is a very simple measure; therefore, it should be part of a broader and more accurate evaluation of patients’ QoL.

### 3.2 HF‐specific measures of QoL

Several issues about the *KCCQ* have been raised. The self‐efficacy subscale seems to have a low reliability (internal consistency). Two items (‘How sure are you that you know what to do, or whom to call, if your HF gets worse?’ and ‘How well do you understand what things you are able to do to keep your HF from getting worse?’) are potentially misleading metrics of self‐efficacy. Indeed, these questions measure disease knowledge rather than operational actions such as liquid restriction or low‐sodium diet. Furthermore, several items (‘How much has your HF limited your enjoyment of life?’, ‘If you had to spend the rest of your life with HF as it is now, how would you feel about this?’ and ‘How often have you felt discouraged because of your HF?’) are a bit redundant and could be eliminated without affecting the questionnaire validity. Conversely, 2 items assessing independence in dressing and bathing could be enucleated as a subscale assessing independent care.^40^

### 3.3 Neuropathy‐specific measures of QoL

The *PND score* has only 6 discrete categories, which do not allow to accurately characterize the patient's clinical status, and to capture a deterioration in neurological function. Indeed, it can take up to 5 years for patients with amyloidosis to transition from one score to another.^36^, ^41^

The *NIS* and its variant, the *NIS+7*, may not be the ideal tool to evaluate patients with early‐stage ATTR amyloidosis,^36^, ^42^ and do not take into account dysautonomia.^42^ The *modified NIS+7* scores (mNIS+7_Alnylam_ and mNIS+7_Ionis_) were introduced specifically for these patients, and proved to have a good reproducibility and ability to detect improvements, but require a long evaluation, which hinders their widespread adoption.^36^

The *Kumamoto Neurologic* *Scale* lacks any psychometric analysis. Following its introduction in 1999 in a Japanese study on V30 M ATTRv amyloidosis,^29^ it has been rarely used, probably because of the wider diffusion of the NIS and its variants.

The *FAP‐RODS* only explores limitations in physical and social activities, and was developed in Portugal in a population including only subjects with V30 M familial amyloid polyneuropathy; therefore, it might not perform well in patients with other mutations or other amyloid types.^30^, ^42^, ^43^

The *Norfolk* *QoL‐DN* score is strongly correlated with disease stage. Conversely, the *Activities of Daily Living and Small‐Fibre Neuropathy* scores were similar in patients with stage 1 disease and healthy volunteers, and the small‐fibre neuropathy domain performed poorly in differentiating patients with stage 2 or stage 3 disease. Items regarding autonomic neuropathy also showed a low discriminative capacity. Furthermore, the validity of the Norfolk QoL‐DN for ATTR‐CM or ATTR‐PN has not been assessed yet.^32^

The *COMPASS‐31* is a good tool to evaluate dysautonomia,^33^ but it is not sufficient alone to estimate the degree of ATTR‐PN.

## 4 PERSPECTIVES FOR NOVEL QOL MEASURES FOR ATTR AMYLOIDOSIS

Since ATTRwt and ATTRv amyloidosis are highly heterogeneous, different QoL metrics should be available for these 2 conditions and should allow a global evaluation of the burden of cardiac disease, neurologic impairment and other systemic comorbidities. While scales remain the main tools to assess disease severity and outcomes, PROMs have the important advantage of considering the disease from the patient's perspective. The availability of reliable measures of disease burden as experienced by patients can improve patient management and trial design. Development and validation of high‐quality PROMs are complex and time‐consuming requiring not only clinician expertise and experience, but also patient involvement. This collaboration allows to identify which domains and specific items have a major impact on QoL. Different specialists should be involved in questionnaire development. The following step would be to reappraise the questionnaire together with a group of patients large enough to be representative of the different disease phenotypes and severity. Questionnaires for ATTR amyloidosis also need to be short and easy to understand by patients of different age, and cultural and social background. Once developed, the questionnaire should be validated on an independent group of patients, to test its reproducibility (ie the stability of score values when a patient remains clinically stable) and response to HF decompensations.

A recent paper reported the results of semi‐structured interviews to 14 patients with ATTRv amyloidosis, aimed to gain insight on disease manifestations and their impact on functioning, well‐being, work and activities of daily living.^44^ Patients emphasized the influence of symptoms on the ability to walk or use their hands. Half of them had feelings of frustration and disappointment, often associated with symptom progression, further loss of functioning, perceived limited benefit from treatment or need to stop working. The majority of patients reported that ATTRv amyloidosis impacted negatively on their work, social relationships and daily activities.^44^ This study emphasized the importance of a global approach to QoL assessment, focusing on the different domains potentially affected by the disease. However, it did not lead to the creation of a PROM for these patients.

**FIGURE 1:**
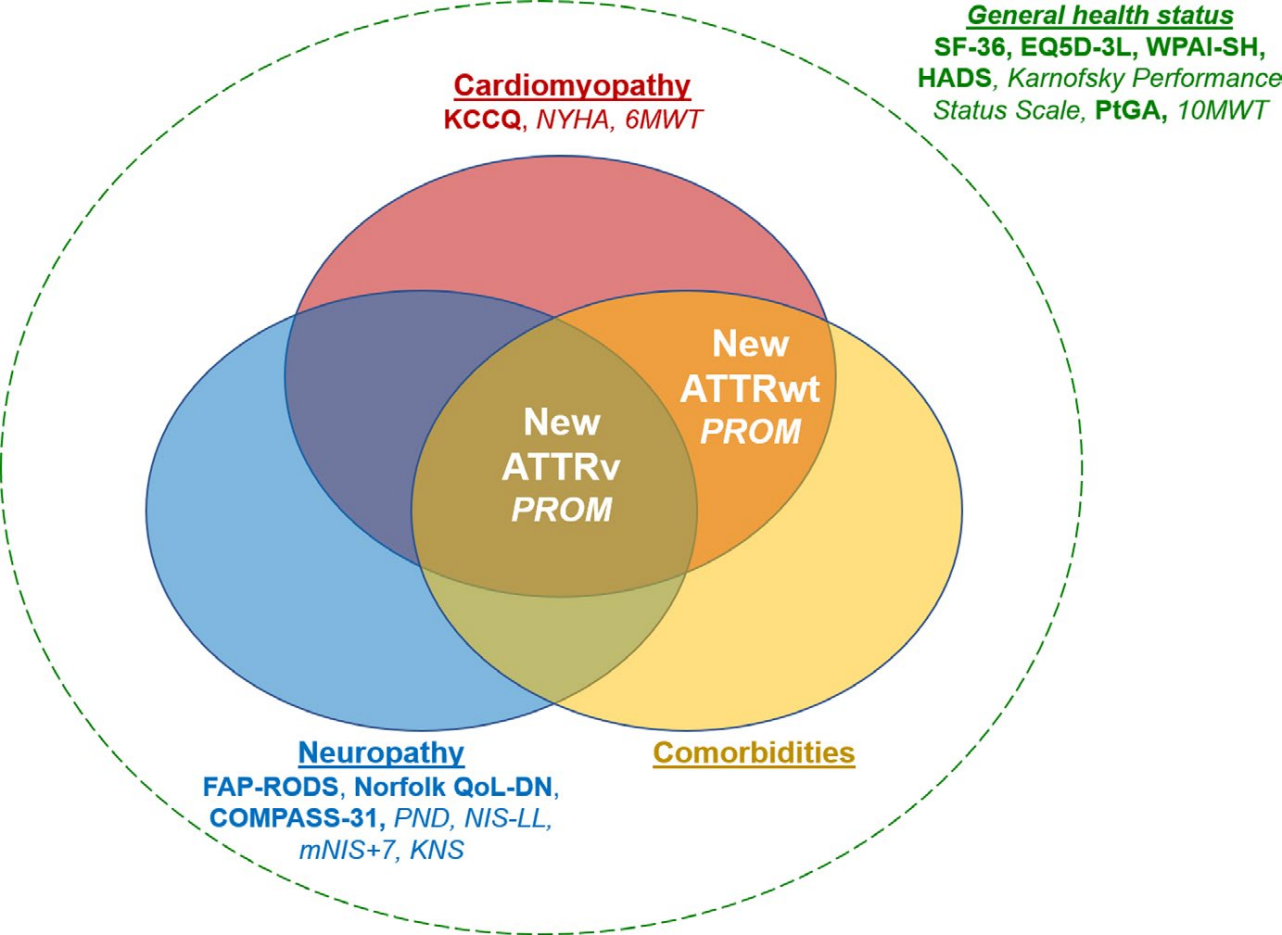
New patient‐reported outcome measures (PROMs) to capture the clinical complexity of variant and wild‐type amyloid transthyretin amyloidosis (ATTRv/ATTRwt). See text for details. Scales and PROMs are reported in italic and in bold, respectively. 6MWT, 6‐min walking test; 10MWT, 10‐metre walking test; COMPASS‐31, COMPosite Autonomic Symptom Scale 31; EQ5D‐3L, Euro QoL 5‐Dimensions 3‐Levels; FAP‐RODS, Familial Amyloid Polyneuropathy specific Rasch‐built Overall Disability Scale; HADS, Hospital Anxiety and Depression Scale; KCCQ, Kansas City Cardiomyopathy Questionnaire; KNS, Kumamoto Neurologic Scale; mNIS+7, modified Neuropathy Impairment Score +7; Norfolk QoL‐DN, Norfolk Quality of Life‐Diabetic Neuropathy questionnaire; NYHA, New York Heart Association; PND, Polyneuropathy Disability Score**;** SF‐36, Short Form 36 Health Survey; WPAI‐SH, Work Productivity and Activity Impairment Questionnaire: Specific Health Problem

To our knowledge, the ITALY (Impact of Transthyretin Amyloidosis on Life qualitY) study (NCT04563286) is the only ongoing trial aiming to develop and validate 2 specific PROMs for ATTRwt and ATTRv amyloidosis, the latter encompassing the whole phenotypic spectrum of this condition, from cardiac‐specific to pure neurological manifestations (Figure 1). This study involves 5 tertiary referral centres for ATTR‐CA in Italy (Pisa, Bologna, Pavia, Firenze and Messina) and aims to enrol at least 250 consecutive patients (50 per centre, with at least 40% of patients with ATTRv amyloidosis). Following a critical revision of medical literature on QoL metrics currently used in ATTR amyloidosis, a panel of cardiologists, internal medicine specialists, neurologists, rare disease specialists, geriatricians and health management specialists selected the most clinically relevant domains for patients with ATTRwt or ATTRv amyloidosis independently. They then chose 10 items for each domain. Afterwards, 2 groups of 25 patients with ATTRwt or ATTRv amyloidosis were selected trying to recapitulate the full spectrum of these conditions, including the cardiac, neurologic and mixed phenotypes of ATTRv amyloidosis. Patients were asked to grade the relevance of each item from 1 to 10. In this way, the 30 most relevant items for ATTRwt or ATTRv amyloidosis were identified. A question was created for each item, resulting in 2 sets of 30 questions with 5 possible answers. Questions and answers were created and formatted for gender neutrality, clarity and interpretability, with the perspective of a future translation from Italian into English and other languages. The questionnaires are enclosed in the Appendix S1. The study is ongoing, with 50 patients already enrolled. At study entry, patients are asked to fill the new PROM and the SF‐36 and the KCCQ. Their NYHA class, 6‐minute walking distance, N‐terminal pro‐B natriuretic peptide and high‐sensitivity troponin are also determined, and patients undergo a transthoracic echocardiogram. Patients are then classified according to the occurrence of HF hospitalization over 6 months. Patients who are hospitalized for HF enter the responsiveness cohort and repeat the baseline examinations at the time of hospitalization. Conversely, patients who are not hospitalized for 6 months after enrolment are re‐evaluated at 6 months and enter the reliability cohort. The goal is to assess whether score values change during an HF hospitalization or display limited variations when patients remain clinically stable. The same design was used to develop the KCCQ.^24^ Score values at baseline and their changes over time will also be compared with the SF‐36, the KCCQ, NYHA class, circulating biomarkers and echocardiographic findings.

The ITALY study is expected to provide validated PROMs specific for ATTRwt and ATTRv amyloidosis, which may represent useful tools for patient management and novel surrogate end points for clinical trials.

## CONFLICT OF INTEREST

None.

## Supporting information

App S1
