# Supplementary material for: Quality of life assessment in amyloid transthyretin (ATTR) amyloidosis
Source: Eur J Clin Invest. 2021 May 22;51(11):e13598. doi: 10.1111/eci.13598 (PMC8596396; doi:10.1111/eci.13598)
Supplement: Supplementary file 1 — App S1 [file ECI-51-e13598-s001.docx]

**Appendix S1**

**ATTRwt questionnaire**

*Dear Patient, the following questionnaire refers to your heart disease and how it affects your quality of life. We ask you to read the following* ***30* *questions*** *carefully and answer each of them by choosing the option that most closely reflects your condition.* Please ***refer to the last 2 weeks.*** *Please remember that there are no right or wrong answers.*

1) How breathless have you felt doing the following activities in the last 2 weeks?

…climbing 2 floors of stairs without stopping

No breathless at all 0

Slightly breathless 1

Mildly breathless 2

Moderately breathless 3

Severely breathless 4

*Not assessable (if bedbound or with severely impaired motility) X*

2) How breathless have you felt doing the following activities in the last 2 weeks?

…running to catch the bus

No breathless at all 0

Slightly breathless 1

Mildly breathless 2

Moderately breathless 3

Severely breathless 4

*Not assessable (if bedbound or with severely impaired motility) X*

3) How breathless have you felt doing the following activities in the last 2 weeks?

…walking at a regular pace for 30 minutes without stopping

No breathless at all 0

Slightly breathless 1

Mildly breathless 2

Moderately breathless 3

Severely breathless 4

*Not assessable (if bedbound or with severely impaired motility) X*

4) How breathless have you felt doing the following activities in the last 2 weeks?

…moving around in your house

No breathless at all 0

Slightly breathless 1

Mildly breathless 2

Moderately breathless 3

Severely breathless 4

5) How often have you avoided doing something due to shortness of breath?

Never 0

Less than twice a week 1

More than twice a week 2

Most days 3

Daily 4

6) How breathless have you felt while lying down with only one pillow?

No breathless at all 0

Slightly breathless 1

Mildly breathless 2

Moderately breathless 3

Severely breathless 4

7) How often have you woken up overnight with a feeling of lack of air?

Never 0

Less than twice a week 1

More than twice a week 2

Most days 3

Daily 4

8) How often have you noticed swelling in feet, ankles or legs in the evening?

Never 0

Less than twice a week 1

More than twice a week 2

Most days 3

Daily 4

9) Do you think you have gained weight?

Definitely not 0

I don’t think so 1

I am not sure 2

Maybe yes 3

Definitely yes 4

10) How often have you felt tired?

Never 0

Less than twice a week 1

More than twice a week 2

Most days 3

Daily 4

11) How often have you felt so tired to ask for help in your everyday activities?

Never 0

Less than twice a week 1

More than twice a week 2

Most days 3

Daily 4

12) How have the heart disease symptoms and signs (breathlessness, swelling in feet, ankles or legs, weight gain, tiredness) changed in the last 2 weeks?

Absence or improvement of symptoms/signs of heart disease 0

Stable symptoms/signs 1

Mildly worsened 2

Moderately worsened 3

Severely worsened 4

13) How sad or depressed have you felt?

Not at all 0

A little 1

Considerably 2

Much 3

Very much 4

14) How unhappy have you felt?

Not at all 0

A little 1

Considerably 2

Much 3

Very much 4

15) How alone have you felt?

Not at all 0

A little 1

Considerably 2

Much 3

Very much 4

16) How worried are you about your heart disease?

Not at all 0

A little 1

Considerably 2

Much 3

Very much 4

17) How worried are you about losing your physical autonomy?

Not at all 0

A little 1

Considerably 2

Much 3

Very much 4

18) If someone told you that your current disease condition would remain stable over time, how would you feel?

Extremely unhappy 4

A little unhappy 3

Neither happy nor unhappy 2

Happy 1

Very happy 0

19) To what extent do you think the course of your disease may be modifiable?

Not at all 0

A little 1

Considerably 2

Much 3

Very much 4

20) Are you worried that your condition may get worse?

Not at all 0

A little 1

Considerably 2

Much 3

Very much 4

21) How relevant is amyloidosis to you compared to other health problems?

Not at all/Minimally 0

A little 1

Considerably 2

Much 3

Very much 4

22) Do you feel capable of managing examinations, therapies, check-ups for your disease?

Not capable at all 4

Slightly capable 3

Mildly capable 2

Moderately capable 1

Absolutely capable 0

23) If you developed health problems, how ready would you feel to manage them?

Not ready at all 4

Slightly ready 3

Mildly ready 2

Moderately ready 1

Absolutely ready 0

24) How worried are you about the negative consequences of examinations and therapies for amyloidosis?

Not worried at all 0

Slightly worried 1

Mildly worried 2

Moderately worried 3

Severely worried 4

25) To what extent do you think cardiac amyloidosis affects your quality of life?

Not at all 0

A little 1

Considerably 2

Much 3

Very much 4

26) To what extent amyloidosis has influenced the relationship with your closest family member (wife, husband...)?

Not at all 0

A little 1

Considerably 2

Much 3

Very much 4

27) How dependent do you feel on others because of your heart disease?

Not at all 0

A little 1

Considerably 2

Much 3

Very much 4

28) Are you concerned that family members may develop the disease?

Not at all 0

A little 1

Considerably 2

Much 3

Very much 4

29) Are you actively involved by your family/friends/neighbours in daily life activities (e.g., convivial dinners/council meetings/family decisions)?

Not at all 0

A little 1

Considerably 2

Much 3

Very much 4

30) In your opinion, how much does the society care about patients with cardiac amyloidosis like you?

Not at all 4

A little 3

Considerably 2

Much 1

Very much 0

*Did someone help you to fill out this questionnaire? Yes No*

***Thanks for participating***

**ATTRv questionnaire**

*Dear Patient, the following questionnaire refers to your heart disease and how it affects your quality of life. We ask you to read the following* ***30* *questions*** *carefully and answer each of them by choosing the option that most closely reflects your condition.* ***Please refer to the last 2 weeks.*** *Please remember that there are no right or wrong answers.*

1) How breathless have you felt climbing 2 floors of stairs without stopping?

No breathless at all 0

Slightly breathless 1

Mildly breathless 2

Moderately breathless 3

Severely breathless 4

*Not assessable (if bedbound or with severely impaired motility) X*

2) How often have you avoided doing something due to shortness of breath?

Never 0

Less than twice a week 1

More than twice a week 2

Most days 3

Daily 4

3) How breathless have you felt while lying down with only one pillow?

No breathless at all 0

Slightly breathless 1

Mildly breathless 2

Moderately breathless 3

Severely breathless 4

4) How often have you noticed swelling in feet, ankles or legs in the evening?

Never 0

Less than twice a week 1

More than twice a week 2

Most days 3

Daily 4

5) How limited do you feel by tiredness?

Not at all 0

A little 1

Considerably 2

Much 3

Very much 4

6) How often have you avoided doing something because of tiredness?

Never 0

Less than twice a week 1

More than twice a week 2

Most days 3

Daily 4

7) How have the heart disease symptoms and signs (breathlessness, swelling in feet, ankles or legs, weight gain, tiredness) changed in the last 2 weeks?

Absence or improvement of symptoms/signs of heart disease 0

Stable symptoms/signs 1

Mildly worsened 2

Moderately worsened 3

Severely worsened 4

8) Have you felt limited by the following disorders?

…sensitivity disorders (tingling sensation, pain...)

Not at all 0

A little 1

Considerably 2

Much 3

Very much 4

9) Have you felt limited by the following disorders?

…dizziness when standing up

Not at all 0

A little 1

Considerably 2

Much 3

Very much 4

10) Have you felt limited by the following disorders?

…gastrointestinal disorders (constipation, nausea, vomit, diarrhoea)

Not at all 0

A little 1

Considerably 2

Much 3

Very much 4

11) Have you felt limited by the following disorders?

…urinary disorders (incontinence, urinary retention)

Not at all 0

A little 1

Considerably 2

Much 3

Very much 4

12) Have you felt limited by the following disorders?

…hearing problems (deafness)

Not at all 0

A little 1

Considerably 2

Much 3

Very much 4

13) Has your life changed after you have been diagnosed with amyloidosis?

Not at all 0

A little 1

Considerably 2

Much 3

Very much 4

14) Are you concerned that family members may develop amyloidosis?

Not at all 0

A little 1

Considerably 2

Much 3

Very much 4

15) How sad or depressed do you feel?

Not at all 0

A little 1

Considerably 2

Much 3

Very much 4

16) How unhappy do you feel?

Not at all 0

A little 1

Considerably 2

Much 3

Very much 4

17) How alone do you feel?

Not at all 0

A little 1

Considerably 2

Much 3

Very much 4

18) How worried are you about amyloidosis and specifically your heart disease?

Not at all 0

A little 1

Considerably 2

Much 3

Very much 4

19) If you knew that amyloidosis and specifically your heart disease would remain stable over time, how would you feel?

Extremely unhappy 4

A little unhappy 3

Neither happy nor unhappy 2

Happy 1

Very happy 0

20) How worried are you that amyloidosis and in particular your heart disease may get worse?

Not at all 0

A little 1

Considerably 2

Much 3

Very much 4

21) To what extent do you think the course of your disease may be modifiable?

Not at all 0

A little 1

Considerably 2

Much 3

Very much 4

22) How relevant is amyloidosis to you compared to other health problems?

Any 0

Little 1

Considerably 2

Much 3

Very much 4

23) Do you feel capable to manage examinations, therapies, check-ups for amyloidosis?

Not capable at all 4

Slightly capable 3

Mildly capable 2

Moderately capable 1

Absolutely capable 0

24) If you developed new health problems, how ready would you feel to manage them?

Not at all 4

A little 3

Considerably 2

Much 1

Very much 0

25) How worried are you about the negative consequences of examinations and therapies for amyloidosis?

Not worried at all 0

Slightly worried 1

Mildly worried 2

Moderately worried 3

Severely worried 4

26) To what extent do you think amyloidosis affects your quality of life?

Not at all 0

A little 1

Considerably 2

Much 3

Very much 4

27) To what extent has amyloidosis affected the relationship with your partner (wife/husband), if any?

Not at all 0

A little 1

Considerably 2

Much 3

Very much 4

28) How dependent do you feel on others because of amyloidosis?

Not at all 0

A little 1

Considerably 2

Much 3

Very much 4

29) Do you feel like having an active part in your family and community (for example, are you involved in family decisions, council meetings, social events)?

Not at all 0

A little 1

Considerably 2

Much 3

Very much 4

30) In your opinion, how much does the society care about patients with amyloidosis like you?

Not at all 4

A little 3

Considerably 2

Much 1

Very much 0

*Did someone help you to fill out this questionnaire? Yes No*

***Thanks for participating***
